# Effects of operational disruptions on corrosion of seabed chains and associated microbial community in offshore mooring systems

**DOI:** 10.3389/fmicb.2025.1715587

**Published:** 2026-01-29

**Authors:** Ketil Bernt Sørensen, Laura Tiano, Solfrid Molid, Øystein Gabrielsen, Turid Liengen

**Affiliations:** 1 Danish Technological Institute, Environmental Technology, Taastrup, Denmark; 2 Danish Technological Institute, Environmental Technology, Aarhus C, Denmark; 3 Equinor ASA, Porsgrunn, Norway; 4 Equinor ASA, Trondheim, Norway

**Keywords:** biofilms, calcite deposits, material integrity, MIC, microbiologically influenced corrosion, North Sea, offshore mooring chains, seabed chains

## Abstract

**Introduction:**

The longevity and operational reliability of offshore mooring chains are critical for the safety of floating oil and gas installations. These chains are subjected to harsh marine environments and numerous stress factors, such as microbiologically influenced corrosion (MIC). This study focuses on MIC on seabed chains under different environments: embedded in sediment or lifted into the water column.

**Methods:**

Microbial communities and corrosion rates on seabed chains were studied during periods of normal operation, with the chains placed with one end in the water column and the other end anchored inside the sediment, and resting periods where the entire seabed chain was on the seabed or buried within. Corrosion rates were measured, and deposits of corrosion products and scale on the seabed chains were studied by microbiological and chemical analysis.

**Results:**

During the study period, microbial communities, including groups of potentially MIC-causing Bacteria and Archaea, were present in the scale material deposited on the surface of the seabed chains. Corrosion rates varied significantly both with time and along the length of the seabed chains, but although the corrosion was at least partly ascribed to MIC, there was no obvious correlation between corrosion rates and numbers of microorganisms present in the local deposits.

**Discussion:**

Several biological and chemical mechanisms are discussed in this paper. The data indicates that complex biogeochemical reactions were contributing to the observed corrosion, including several different biological pathways and types of S- and Fe-cycling, formation of protecting mineral layers and distribution of anodic and cathodic sections locally along the length of the seabed chains. Our findings emphasize the dynamic and unpredictable role of microbial communities, driving complex and spatially structured corrosion.

## 1 Introduction

**Figure 1:**
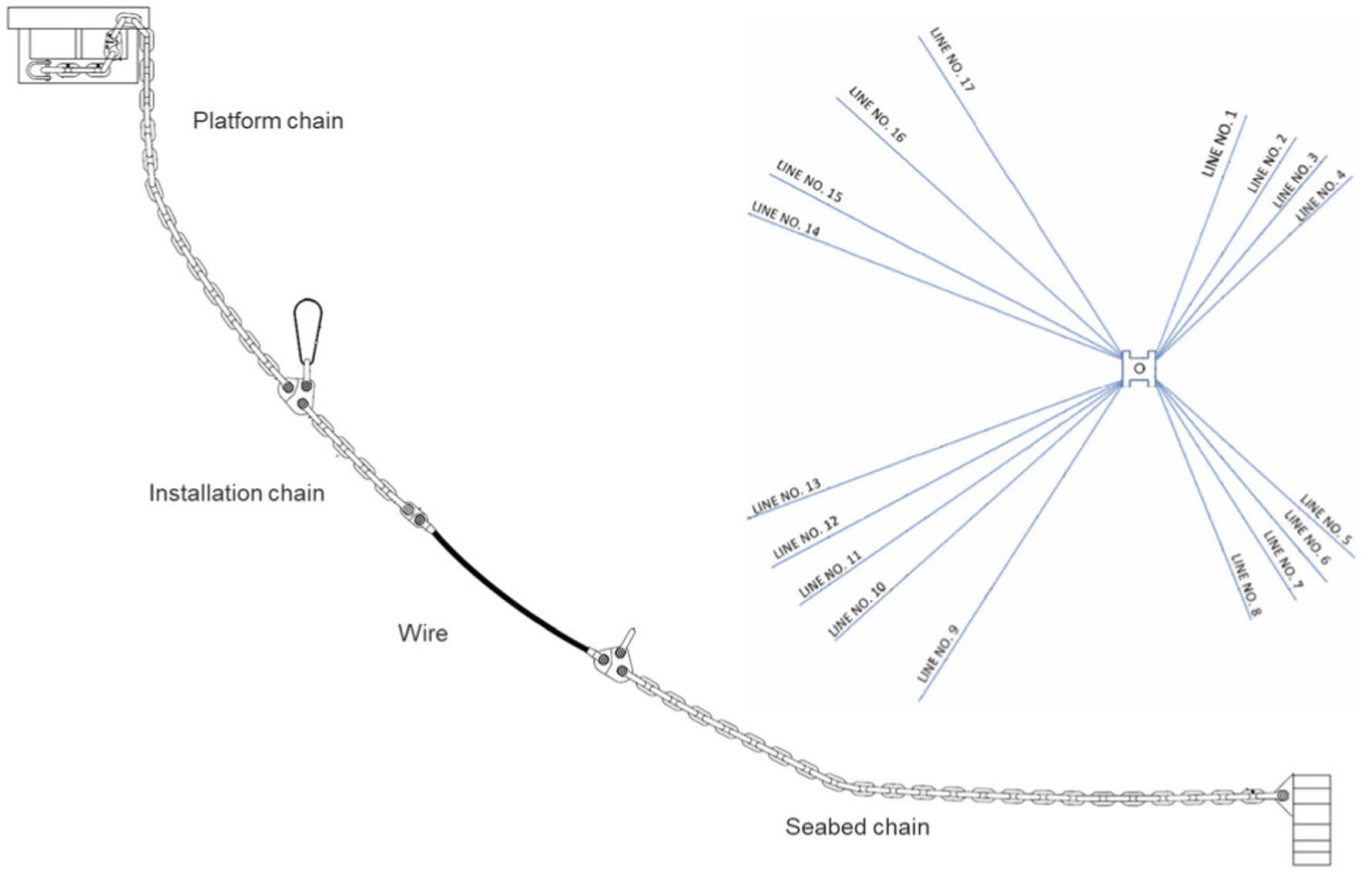
Illustration of a typical mooring line construct, including platform chain, installation chain, wire, and seabed chain. This paper focuses on corrosion and microbial content on the seabed chains.

Marine environments pose significant challenges to offshore mooring systems such as fatigue and corrosion. The latter includes both general corrosion due to oxygen and local corrosion promoted by growth of microorganisms (Liu et al., 2023; Melchers and Lee, 2021), promoting microbial influenced corrosion (MIC) (Melchers and Lee, 2021; Liengen et al., 2014). Mooring lines are used for linking an anchor point to a floating structure, and typically include a platform chain, wire, and seabed chain as illustrated in Figure 1. Typically, the seabed chains are constructed in R4 grade steel containing 0.25% carbon, often referred to as “normal carbon steel.” The seabed chains are interesting study sites of marine corrosion, as different sections are exposed to diverse and varying environmental conditions. The segment of the seabed chain that is closest to the anchoring point is typically permanently embedded in anaerobic sediments, whereas other segments may be exposed to the sediment–water interface, or suspended in the oxygenated water column, either permanently or periodically. Each of these different zones presents unique biogeochemical conditions that influence the type and rate of local corrosion processes (Zhang et al., 2022; Gabrielsen et al., 2018). Furthermore, physical and operational factors such as the movement of seabed chains, periodic lifting or resting, and the absence of protective measures (e.g., anodes) can further modulate corrosion risks and microbial colonization patterns (Zhang et al., 2022; Gier, 2014).

Several studies have documented the potential role of MIC on mooring lines deployed in deep- as well as shallow-water locations (Ma et al., 2013; Fontaine et al., 2014; Zhang et al., 2022; Gabrielsen et al., 2018). Localized corrosion, such as pitting, can be particularly insidious because it may not always result in substantial mass loss but can create stress concentrators and initiate cracks (Liu et al., 2025). This, in turn, accelerates the risk of mechanical failure, especially under the dynamic loading conditions typical of floating installations (Zhang et al., 2022). In recent years, several field studies have reported severe, unexpected corrosion damage and even premature failures of mooring chains attributed largely to MIC, with the phenomenon observed in both deep and shallow waters across different geographic regions (Fontaine et al., 2014; Melchers and Lee, 2021). The effect of MIC on fatigue has been documented on mooring lines (e.g., Gabrielsen et al., 2021), showing the importance of an all-around integrity evaluation for moorings. As many offshore installations dedicated to oil and gas production are aging, it is necessary to reevaluate their lifetime, as well as achieving in-depth understanding of the interaction between the local environment and the MIC rates.

A number of different types of microorganisms such as sulfate-reducing Bacteria (SRB), iron-oxidizing and iron-reducing Bacteria, (IOB and IRB), acid-producing Bacteria (APB) and methanogenic Archaea (MEA) can cause MIC on seabed chains. These microorganisms can colonize steel surfaces and induce or accelerate local corrosion directly or indirectly in a number of ways, e.g., by generating metabolic products such as hydrogen sulfide (H_2_S) and organic acids that can cause localized acidification, depassivation or by harvesting electrons directly from the steel, resulting in release of iron and material loss (Liu et al., 2023; Enning et al., 2012; Lahme et al., 2021). The SCORCH Joint Industry Project (JIP) and related studies have documented that MIC can lead to local corrosion rates up to 10 times higher than general corrosion rates. Depth of local grooves may be sufficient to threaten the structural integrity of anchor chains or other structures, and it might occur well before significant overall material loss is observed (Potts et al., 2012; Fontaine et al., 2014; Zhang et al., 2022).

This study investigates the microbial communities forming on seabed chains, their variation across environmental zones, and operational history of the mooring facility and their impact on corrosion.

## 2 Materials and methods

### 2.1 Field site

The study site is located in the North Sea, where 17 seabed chains of two different lengths (475 and 800 m) are anchored and deployed in all celestial directions (Figure 1). The seabed chains are not protected by anodes and are constructed of R4 grade steel with 0.25% carbon with a diameter of 136 mm. The water depth is approximately 300 m at the facility, and the predominant sediment type is mud containing sand (0.063–2.0 mm grain size) and clay (≤0.002 mm grain size).^1^

The seabed chains investigated in this study were installed in 1997 and operated normally until 2016. During this 19-year period, the anchor ends of the seabed chains were buried in the sediment more than 2 m below the sediment/water interface. At the opposite end, where they met the wire, the seabed chains were positioned in the water. The touchdown point of the seabed chains with the sediment was between 300 and 400 m from the wire. Between the anchoring end and the touch-down point the seabed chains were constantly embedded in the seabed.

Between 2016 and 2022, the seabed chains were out of operation and left on the seabed.

From 2022, the seabed chains were back in normal operation.

**Figure 2:**
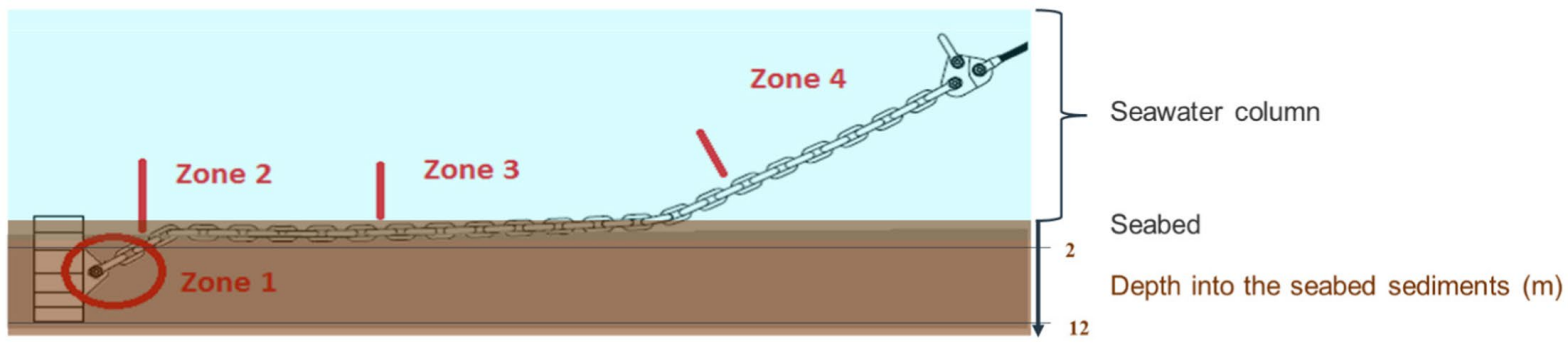
Illustration of the seabed chain and its zones. Zone 1: embedded more than 2 m deep into the sediments, Zone 2: slightly submerged in the sediment in a depth of 0–2 m below the water/sediment interface, Zone 3: the touch down zone, where the chain is moving in and out of the seabed upper layers, Zone 4: always in the water column, (4a) 0–50 m from the wire and (4b) 50–300 m from the wire. In 2019 sampling of deposits was done from all the 4 zones along chain number 1. In 2023 sampling were focused on zone 3 and 4 on chain number 8 and 16.

One of the lines at the facility (Line 12, Figure 1) was retrieved in 2016 and corrosion along the total length of the seabed chain was reported (Gabrielsen et al., 2018). On that occasion, the authors defined the following zonation of the seabed chains with respect to corrosion which will also be used here (illustrated in Figure 2):

**Zone 1:** Near the anchor where the chain is embedded more than 2 m below the sediment surface. In 2016, no significant corrosion was found in this zone.

**Zone 2:** Further away from the anchoring point but permanently embedded in the seabed, i.e., more than 400 m from the wire. In this zone, signs of local corrosion, probably caused by MIC, were detected in the 2016 study.

**Zone 3:** Touchdown zone (between 300 and 400 m from the wire) where the seabed chain is either lying in the upper sediment layers or slightly lifted out of the sediment. No significant corrosion was found in this area in 2016.

**Zone 4:** This part of the seabed chain is 0–300 m from the wire and thus positioned permanently in the seawater. In 2016, general corrosion due to oxygen as well as local corrosion due to MIC caused by SRB was reported from this zone. In this study, zone 4 is further divided into subzone 4a (0–50 m from the wire) and 4b (50–300 m from the wire).

### 2.2 Samples

**Figure 3:**
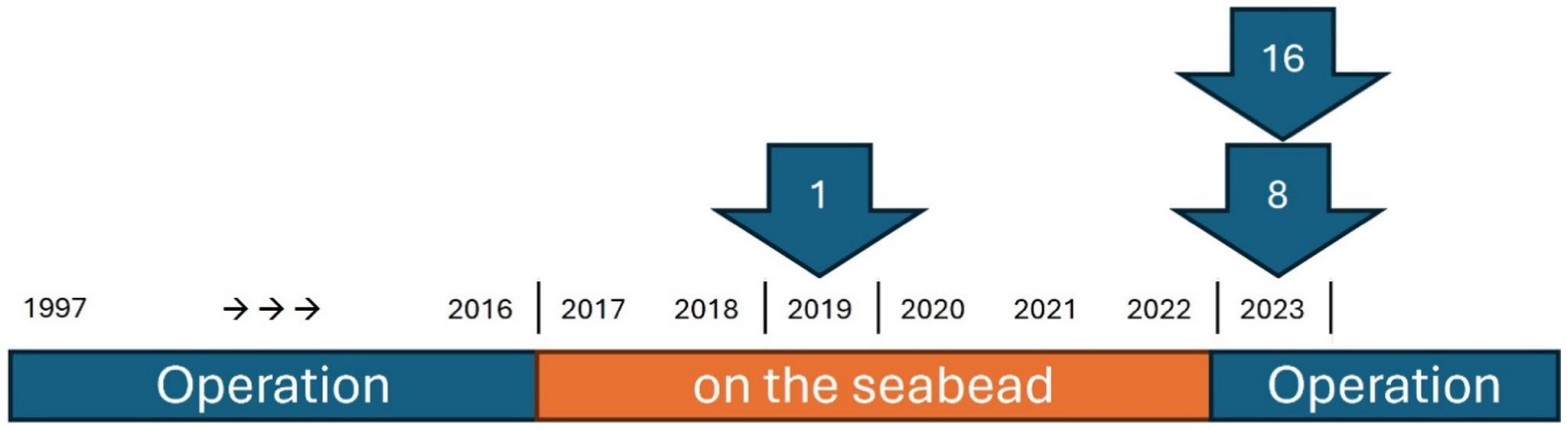
Illustration of the operational history and sampling time of the seabed chains. The arrows indicate the chain number and the year in which it was retrieved.

**Figure 4:**
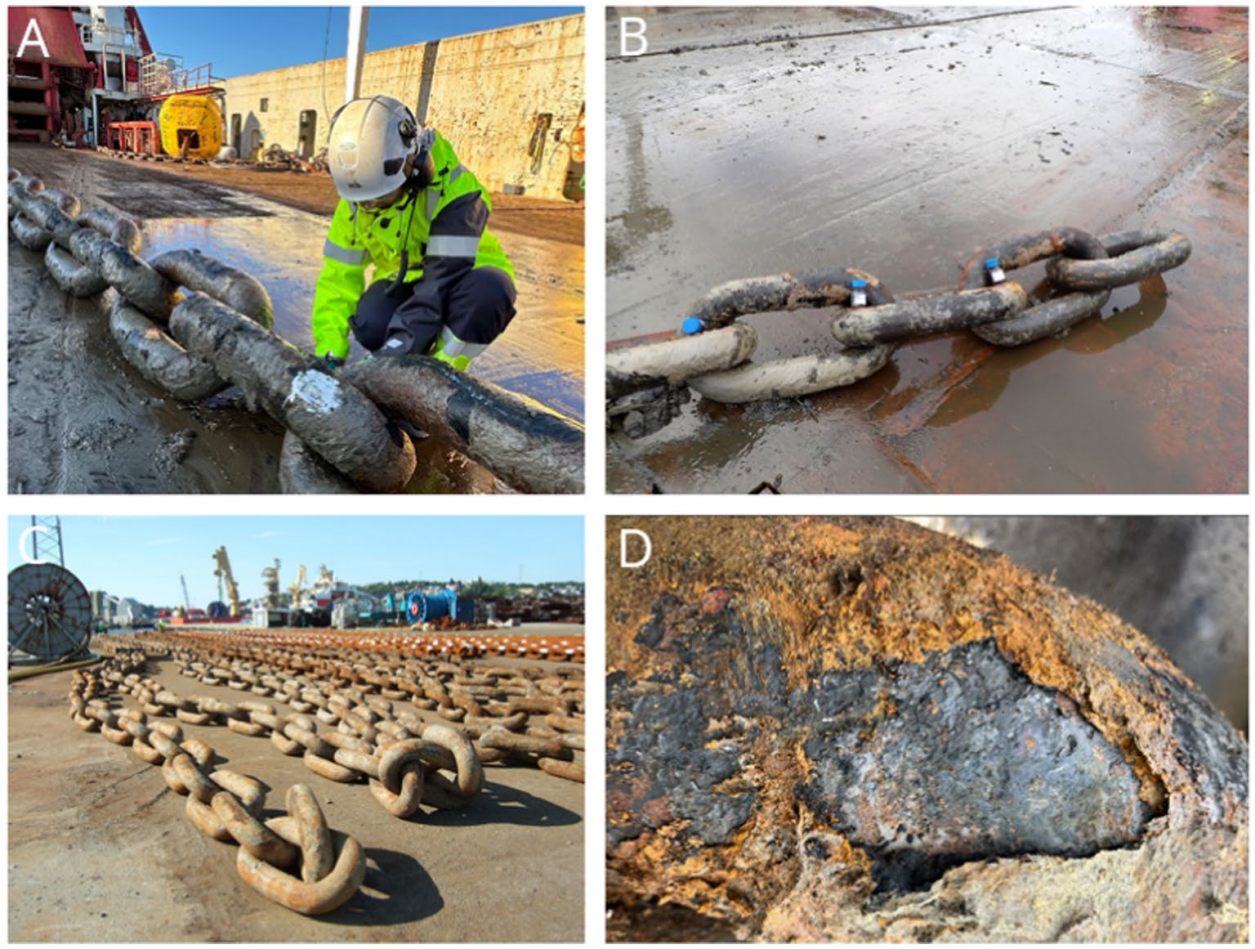
Photos taken during sampling. Panels **(A,B)** illustrate the state of the seabed chains during offshore sampling, immediately after retrieval, when they were still moist with seawater and black and gray in color. Photos shown in panels **(C,D)** were taken during sampling onshore, where the seabed chains were dried out on the surface and the deposits had changed color to rust red due to oxidation with atmospheric air. Photo: Aminda Ripe and personnel from Global Maritim.

The seabed chains and the samples collected for this study are summarized in Table 1 and Figure 3. Sampling offshore took place right after retrieval of the seabed chain, the chain was still wet and the deposits were colored black and gray as shown in Figure 4, panels A and B. Deposit samples scraped from the chain surfaces were collected in 100 mL glass bottles, with as much deposit as possible per bottle. Water samples were collected by an ROV (remotely operated vehicle), collecting water at 20-m depth and ~1 m above the seabed. The ROV collected 1 liter of water. This water was transferred to 100 mL glass bottles, and the bottles were filled to the rim. The deposit samples and the water samples were kept in a refrigerator until the samples were shipped to the laboratory for analysis. The deposit samples were analyzed with respect to microbial content and inorganic compounds. The water samples were analyzed with respect to sulfate and nutrient content.

**Table 1:** Summary of seabed chains and samples retrieved during this study.

Line no.	Retrieved in	Operational history	Zones retrieved	Samples and analyses
1	2019	Normal operation for 19 years and subsequently left unused for 3 years on the seabed.	All zones	Deposits were sampled offshore on the boat retrieving the mooring from all zones for XRD/XRF analysis and microbiological characterization. Water samples were taken by a remotely operated vehicle (ROV) about 1 meter above the seabed and at 20 m water depth for analyses of sulfate and bacterial nutrients. A seabed sediment sample was taken by ROV as a reference sample for the microbial characterization Deposits were sampled onshore for XRD/XRF and SEM/EDS
8	2023	Normal operation for 19 years followed by 6 years on the seabed and finally 1 years in normal operation.	Zone 4	The same samples as in 2019, but deposits were just sampled offshore from Zone 4a and 4b
16	2023	Normal operation for 19 years followed by 6 years on the seabed and finally 1 years in normal operation.	Zone 4	The same samples as in 2019, but deposits were just sampled offshore from Zone 4a and 4b

The seabed chains were then transported onshore where new sampling took place. The transport to the quay took 2–3 days. The chains had then dried and the outer layer of corrosion products had been oxidized as shown in Figure 4, panels C and D. Deposit samples were scraped and hammered off the surface of the chain links. Deposits were collected in plastic vials, one vial per link sampled from. The plastic vials were shipped back to the laboratory within 24 h after sampling. The plastic vials were then stored without lid in a desiccator until analyses of inorganic compound by XRD and XRF. SEM/EDS were carried out on tubercles or flakes of deposits from along the seabed chain.

In 2019 sampling of deposits was done from all the 4 zones along chain number 1. In 2023 sampling were focused on zone 3 and 4 on chain number 8 and 16.

### 2.3 Corrosion measurements

Visual inspection and sampling from the seabed chains were done after placement of the chains at quay. Depth of individual, local corrosion attacks were measured using the depth rod on the end of a caliper. The accuracy of the measurements were ±0.2 mm. The maximum local corrosion rate was calculated by dividing the maximum depth of the local corrosion attacks by years in operation. In order to calculate maximum general corrosion, the remaining diameter of individual chain links were measured using a big size caliper. The maximum general corrosion rate was calculated as difference between original diameter and the measured diameter divided by 2 and then divided by number of years in operation (mm/year).

### 2.4 Chemical analysis

Deposit samples were analyzed by XRD (X-ray diffraction, PANalytical X’pert PRO, Programe Highscore Plus and Datacollector, database ICDD PDF-22016, scan start angel 5.003, end angle 60.000°, step size 0.0167113°, time per step 34.925 s, scan speed 0.060768°/s, preset counts 10,000 counts, number of steps 3,291, runtime 00:16:00, generator MPPC, sample stage Spinner PW3064) and XRF (X-ray fluorescence, PANalytical Axios instrument), to investigate the presence of corrosion products and other inorganic deposits on the surface of the mooring lines. Identification of crystalline compounds present in the deposit samples were done by XRD. XRF data were used to give an estimate of the amounts of different compounds.

Scanning electron microscopy (SEM, Jeol, JSM-IT700HR) and energy dispersive X-ray spectroscopy (EDS) were also used to analyze the inorganic deposits from the mooring lines. SEM mapping signal BED-C, landing voltage 15.0 kV, WD 10.1 mm, magnification ×30, vacuum mod low vacuum (30 kPa), Std-PC 90.0. SEM/EDS Accelerating voltage of 150 kV. SEM/EDS elemental analysis signal BED-S, landing voltage 20.0 kV, WD 10.2, magnification ×100, FOV 1.280 mm × 0.960 mm, vacuum mode low vacuum (30 kPa), probe current number Std 71, scan rotation 48.9°.

Sulfate and microbial nutrients were analyzed by ion chromatography (Thermo Scientific Dionex IC-6000) and total organic carbon was analyzed using a TOC analyzer (Shimadzu TOC-L CPH).

### 2.5 Microbiological analysis

**Figure 5:**
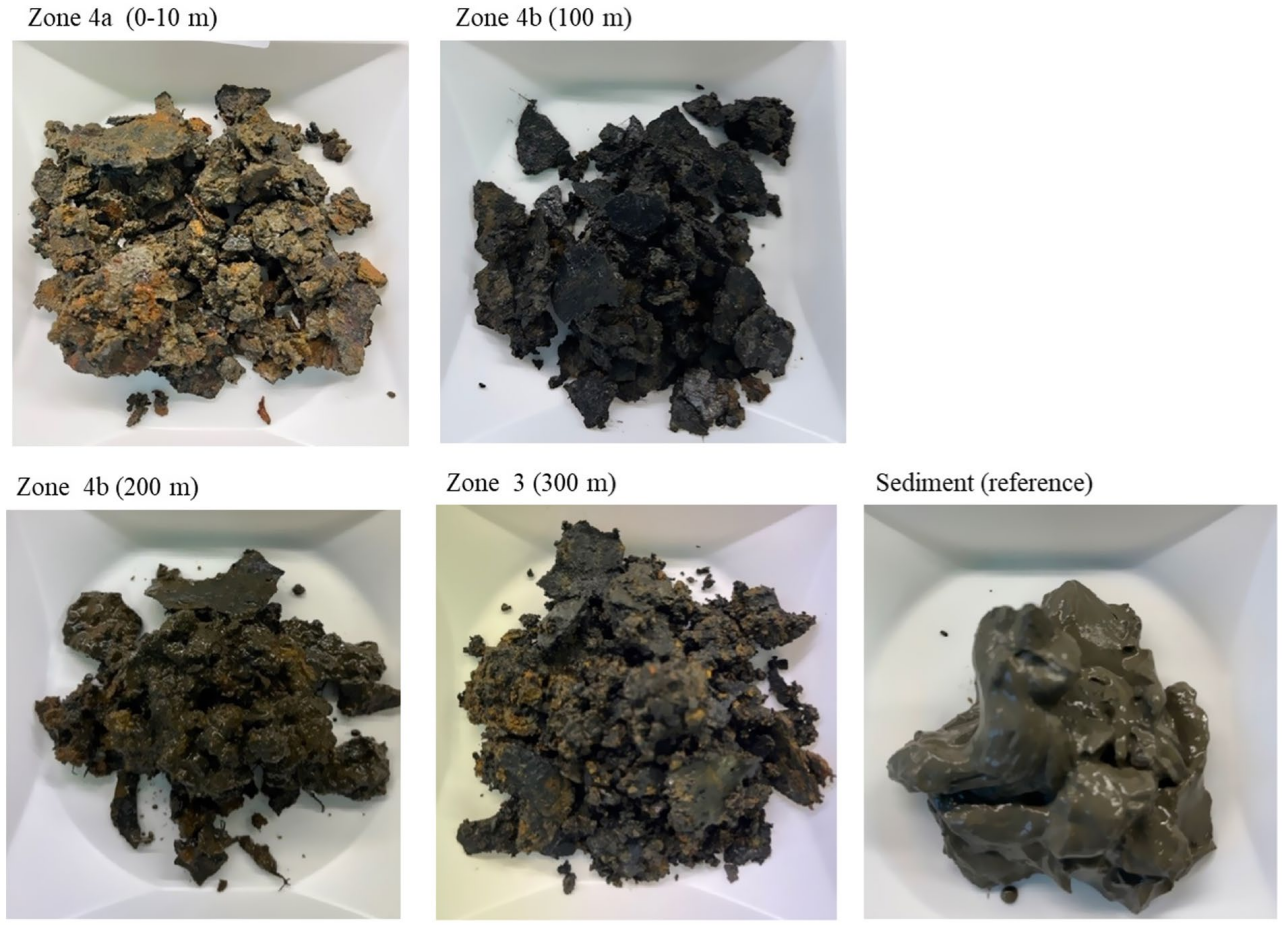
Samples of deposits from seabed chains 8, sampled in 2023 right after retrieval of the seabed chains and used for microbiological analysis.

**Figure 6:**
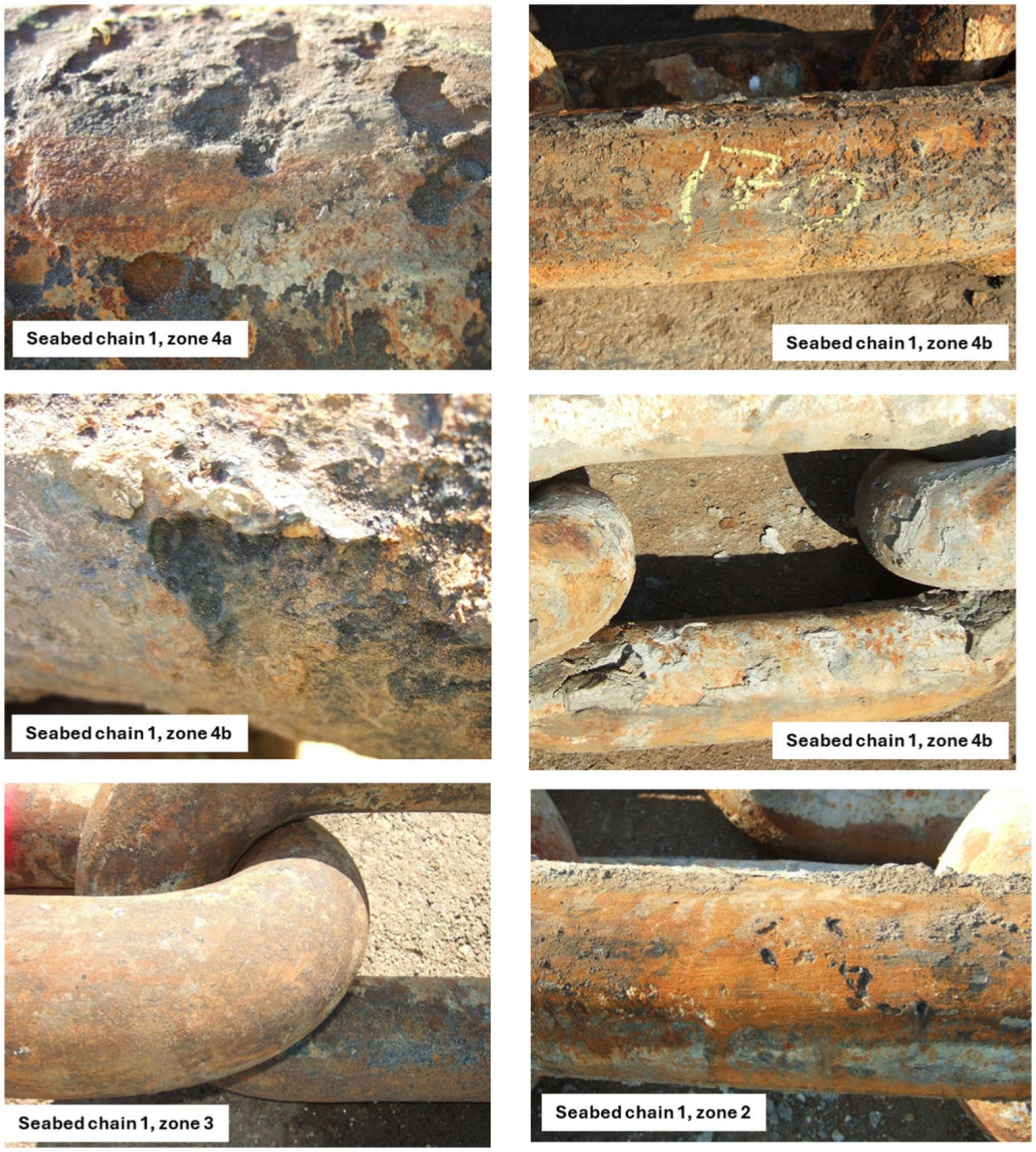
Photos of seabed chain no. 1 retrieved in 2019. The original diameter of the chain links are 136 mm, and the original length of each link is 816 mm.

Samples of deposits from the different zones of the seabed chains 1, 8, and 16 were collected after retrieval of the anchor chain (Figures 5, 6). The samples were homogenized, and subsamples of about 1 g were weighed and used in further analysis. DNA was extracted from the samples, and the numbers of *Bacteria* and *Archaea* were quantified by qPCR targeting 16S rRNA genes (16S rDNA) as described in Gittel et al. (2009). The microbial communities were characterized phylogenetically by amplicon sequencing of 16S rDNA using an Oxford Nanopore Minion according to the manufacturer’s recommendations. The phylogenetic data were then translated into functional groups by considering the most likely metabolisms of closely related organisms in the SILVA database.

## 3 Results

### 3.1 Corrosion rates

Pictures of links from seabed line no. 1 retrieved in 2019 and Line no. 16 retrieved in 2023 are shown in Figure 6. The results of the corrosion measuremernts are listed in Table 2 together with data from 2016 (Gabrielsen et al., 2018). Corrosion rates calculated based on the data from Table 2 during the periods 1997–2016, 2016–2019 and 2019–2023 are given in Table 3.

**Table 2:** Maximum depth of corrosion attacks measured along the seabed chains retrieved in 2016, 2019 and 2023.

Zones on the bottom chain	Maximum depth of corrosion attacks (mm)		
	2016^*^	2019	2023
Zone 4a: 0–50 m from the wire	1	5	5
Zone 4b: 50–300 m from the wire		2	3
Zone 3: Touch down. 300–400 m from the wire	~0	1	~0
Zone 2: More than 400 m from wire. Always in the seabed sediments	4	~0	–
Zone 1: Last links against the suction anchor. More than 2 m into the seabed sediments	~0	~0	–

Uncertainty is ±0.2 mm.

* Gabrielsen et al. (2018).

**Table 3:** Corrosion rates measured along the seabed chains retrieved in 2016, 2019 and 2023.

Zones on the bottom chain	Corrosion rate (mm/year)		
	1997–2016^*^	2016–2019	2019–2023
Zone 4a: 0–50 m from the wire	<0.05	≤1.3	~0
Zone 4b: 50–300 m from the wire		≤0.3	≤0.3
Zone 3: Touch down. 300–400 m from the wire	~0	≤0.04 (1997–2019) ≤0.3 (2016–2019)	~0
Zone 2: More than 400 m from wire. Always in the seabed sediments	<0.2	~0	–
Zone 1: Last links against the suction anchor. More than 2 m into the seabed sediments	~0	~0	–

* Gabrielsen et al. (2018).

No significant corrosion was observed in Zone 1 on any of the seabed chains investigated in 2016 and 2019. Localized corrosion was observed in Zone 2 of the seabed chain retrieved in 2016, whereas Zone 2 of the seabed chain retrieved in 2019 did not show signs of general or localized corrosion. In Zone 3, minor localized corrosion attacks were observed on the seabed chain retrieved in 2019, but not on the seabed chains retrieved in either 2016 or 2023. In summary, it was assessed that Zone 2 and 3 were subjected to only low rates of localized corrosion throughout the study period from 1997 to 2023.

Looking into the period between 1997 and 2016, Zone 4 of the seabed chain experienced a low rate of general and local corrosion (Gabrielsen et al., 2018). In the subsequent period from 2016 until 2019, while zone 4 of the seabed chain was left on the seabed, local corrosion rates were as high as 1.3 mm/year in zone 4a and 0.3 mm/year in zone 4b. In the final period from 2019 to 2023, with zone 4 of the seabed chains re-positioned in the water column in 2022 the corrosion rate in zone 4a was ~0 mm/year and in zone 4b less than 0.3 mm/year.

### 3.2 Inorganic deposits along the seabed chains

**Figure 7:**
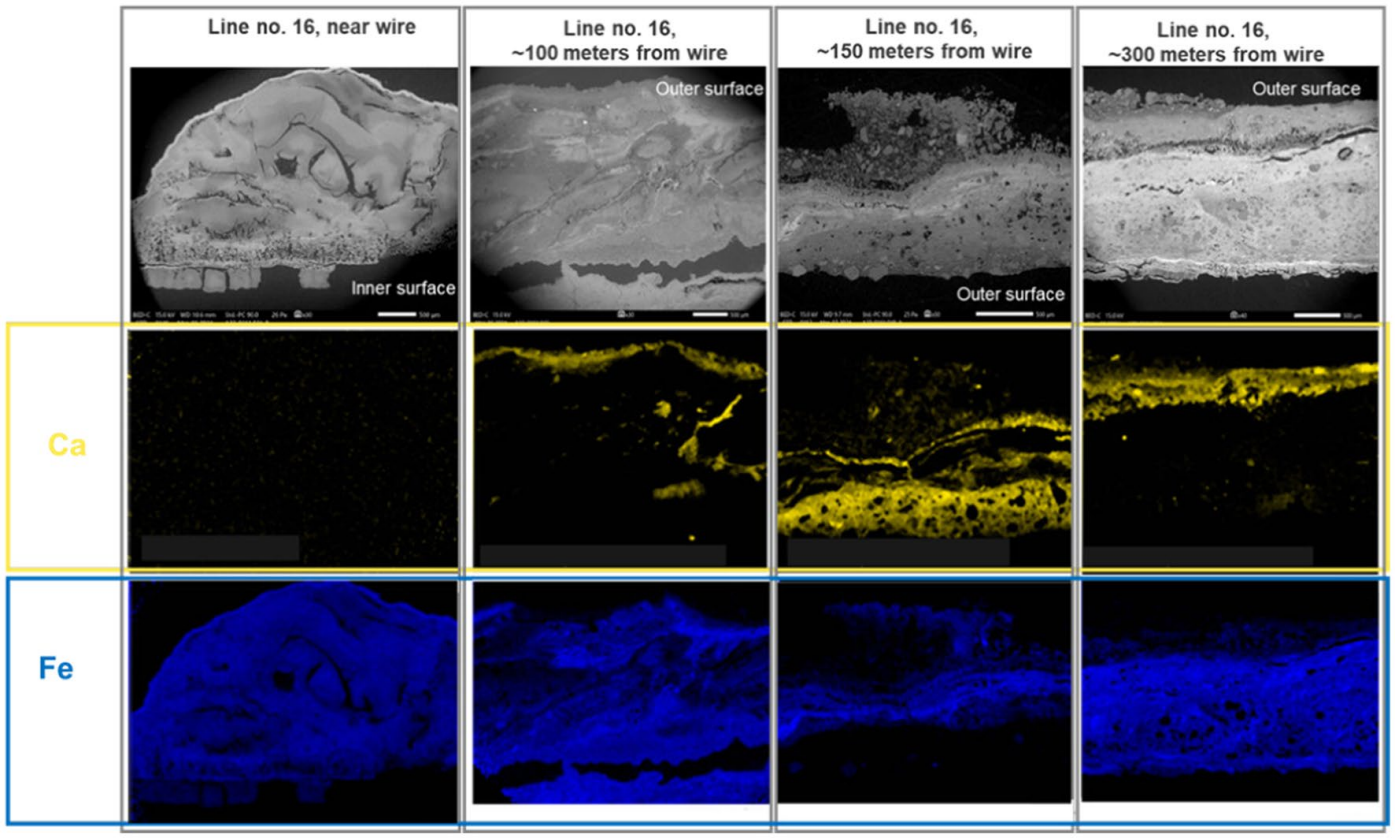
SEM/EDS pictures demonstrating layers containing calcium at the outer surface of the deposits from seabed chain no. 16, zone 4. Scale bar on the SEM pictures at the top is 500 μm.

Results of the XRD/XRF analysis are given in Table 4. Inorganic deposits were dominated by iron-containing minerals, probably corrosion products, and calcite. The former was detected in the form of iron hydroxides (FeOOH), magnetite (Fe_3_O_4_), iron sulfide (FeS), iron carbonate (FeCO_3_), and “green rust,” which is a collective term for various green crystalline chemical compounds containing iron, iron cations, hydroxide anion, and another anion such as carbonate, chloride, or sulfate (Refait et al., 1998). Furthermore, SEM/EDS analysis demonstrated a pronounced spatial pattern, with calcium present mainly in distinct layers at the outer surface of the deposits (Figure 7, middle row of photos), whereas iron was present throughout the deposits (Figure 7, bottom photos), but in lower concentrations where the calcium concentration was highest.

**Table 4:** Inorganic components present in samples from seabed chain no. 1 (ML1) retrieved in 2019 and seabed chain no 8 (ML8) and 16 (ML16) retrieved in 2022 based on XRD and XRF measurements.

Sample name	Zone	Iron containing corrosion products					Scale
		Group 1a Iron hydroxides	Group 1b Magnetite	Group 2			Group 3
		FeO(OH)	Fe_3_O_4_	FeS	FeCO_3_	Green rust	CaCO_3_
The installation							
The wire							
ML8 Link# 1	4a	20–30	5–15	1–5	–	30–40	25–35
ML16 Link# 5–11	4a	60–70	5–15	3–8	5–15	5–10	–
ML 1 Link# 20	4a	–	90–95	5–10	–	–	–
ML16 Link# 165	4b	25–35	3–8	10–15	1–5	25–35	15–25
ML1 Link# 172	4b	20–30	5–10	30–40	10–20	–	15–20
ML8 Link# 211	4b	20–30	25–35	5–15	5–15	5–15	15–25
ML16 Link# 352	4b	15–25	3–8	5–10	1–5	1–5	55–65
ML1 Link”361	4b	50–60	5–10	15–30	15–25	–	10–20
ML8 Link# 375	4b		45–55	5–10		25–35	5–15
ML16 Link# 537	3	30–40	–	1–5	1–5	–	55–65
ML8 Link# 538	3	–	10–20	5–10	5–15	5–15	50–60
ML1 Link# 601	3	–	20–30	20–30	10–15	–	35–45
ML1 Link# 812	2	–	–	10–15	10–20	–	65–80
ML1 Link#846	1	–	–	1–5	50–60	–	35–50
The sediments							

The components are divided into different groups representing corrosion products and scale. Green rust collective term for various green crystalline chemical compounds containing iron, iron cations, hydroxide anion, and another anion as carbonate, chloride, or sulfate. Sediments are left out. The results are given as weight % of the components belonging to each group. Iron sulfide was present in an amorphous form in all the samples. The color code refers to the different zones the samples are taken from.

Comparing the data from 2019 (mooring line no. 1—ML1) with the data from 2023 (ML8 and ML16) in Table 4 indicates several developments: In 2019, after spending 3 years lying on the seabed, the deposits in Zone 4a consisted mainly of magnetite mixed with smaller amounts of iron sulfide. In 2023, after having subsequently been placed in the water column, the deposits from the same zone had become much more complex, with abundant iron hydroxide, magnetite, green rust, as well as some iron sulfide, iron carbonate and calcium carbonate.

Simultaneously, a decrease in iron sulfide content from 2019 to 2023 was found in Zone 4b and Zone 3, as well as an increase in the amount of green rust in Zone 4b. Green rust is considered an unstable intermediate formed as a product of corrosion in low-oxygen environments (Refait et al., 1998), and its presence suggests that a more active biogeochemical iron cycling was occurring in the deposits in 2023 compared to 2019.

Finally, it also appears that the content of calcium carbonate increased from 2019 to 2023. The SEM/EDX analysis clearly showed that most of the CaCO_3_ formed close to the deposit/water interface during this period, probably as a consequence of local pH gradients coupled to biogeochemical S- and Fe-cycling.

### 3.3 Microbial nutrients in seawater

The results of chemical characterization of seawater from the field site are summarized in Table 5.

**Table 5:** Sulfate and nutrients in water samples from the area where the seabed chains were positioned.

Sample name	SO_4_^2−^	NO_3_^−^	NH_4_^+^	PO_4_^3−^	Acetate	Formate	TOC
2019 Line no. 1, 35 m water depth	2,460	<4	<4	0.1	<5	<3	1.4
2019 Line no. 1, 1 m above seafloor	2,520	<4	<1	0.1	<5	<3	250
2023 Line no. 16, 20 m depth	2,610	<3	<1	<0.04	<2	<2	1.9
2023 Line no. 16, 1 m above the seafloor	2,690	<3	<1	0.06	<2	<2	1.3

The results are given as mg/L.

Results indicated low levels of nutrients (N and P) in the water as well as low levels of organic material.

### 3.4 Microbiological analyses

**Figure 8:**
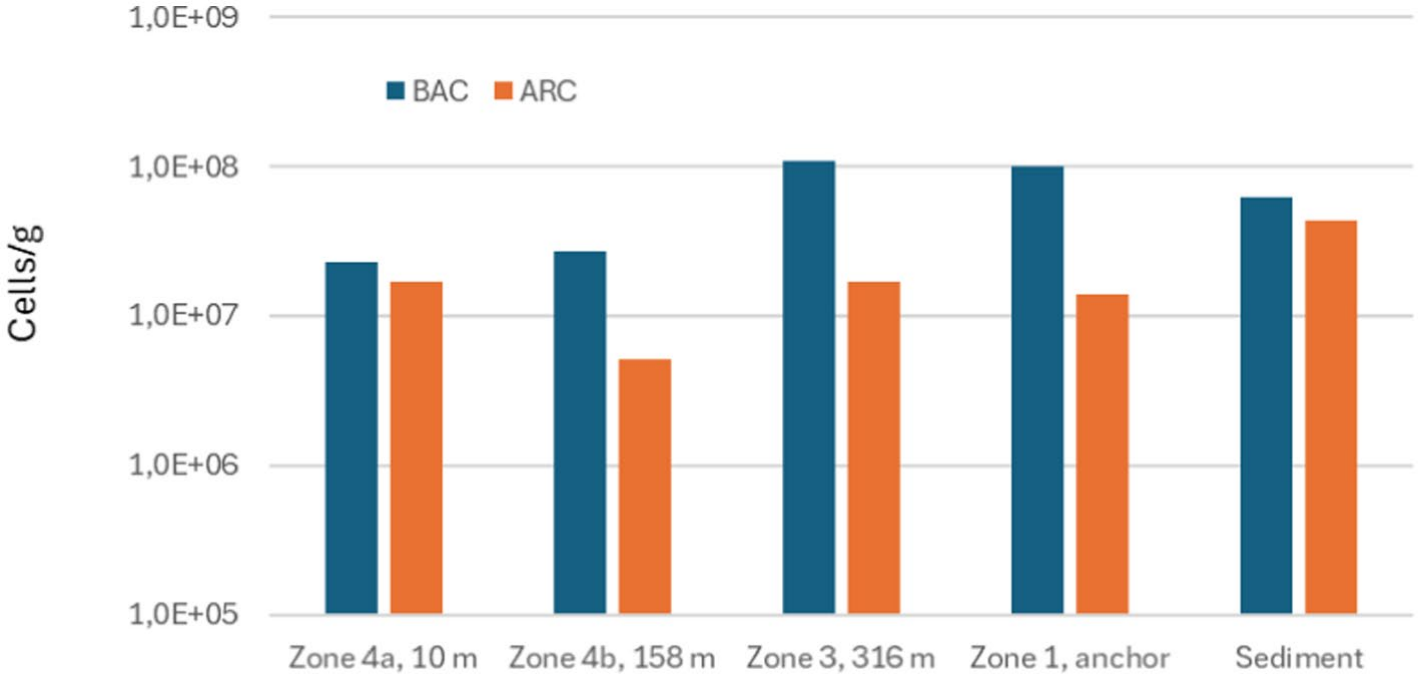
Total numbers of Bacteria (BAC) and Archaea (ARC) measured in scale from different zones on seabed chain 1, sampled in 2019.

**Figure 9:**
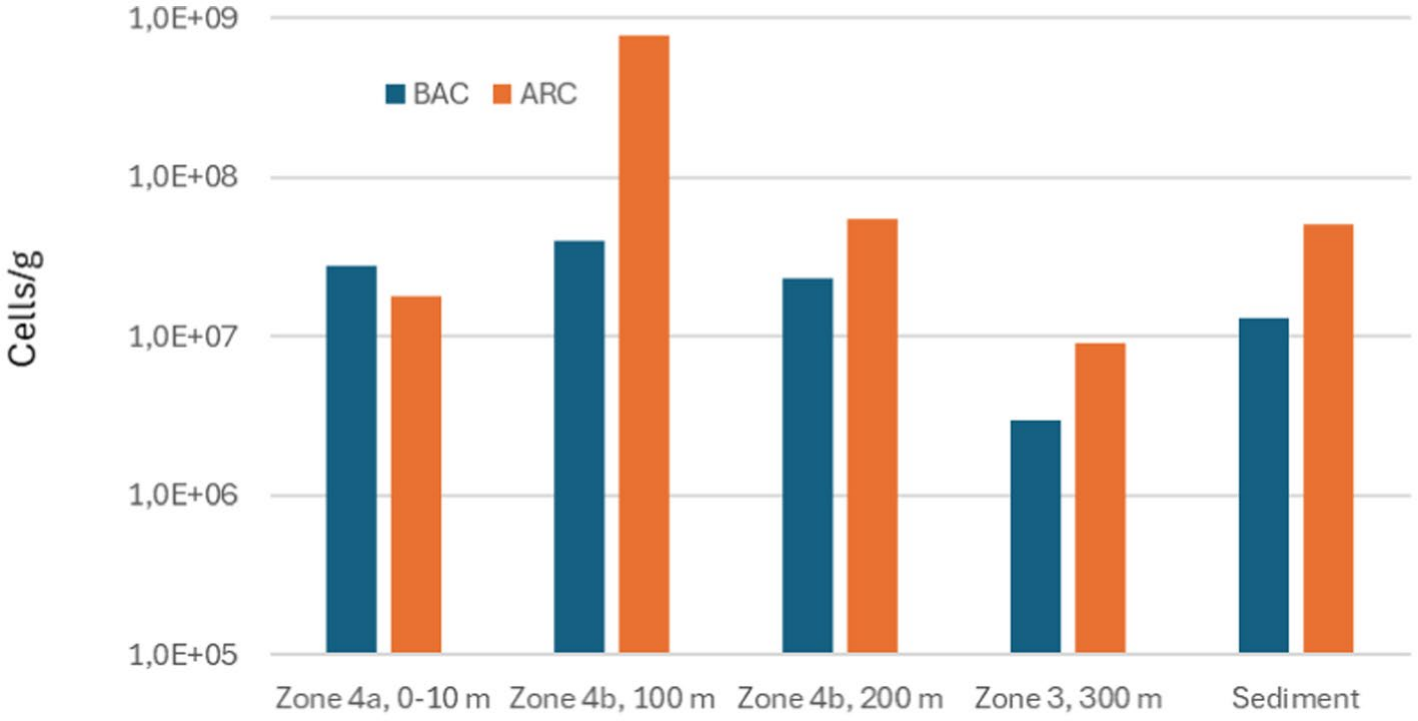
Total numbers of Bacteria (BAC) and Archaea (ARC) measured in scale from different zones on the seabed chain 8, sampled in 2023.

**Figure 10:**
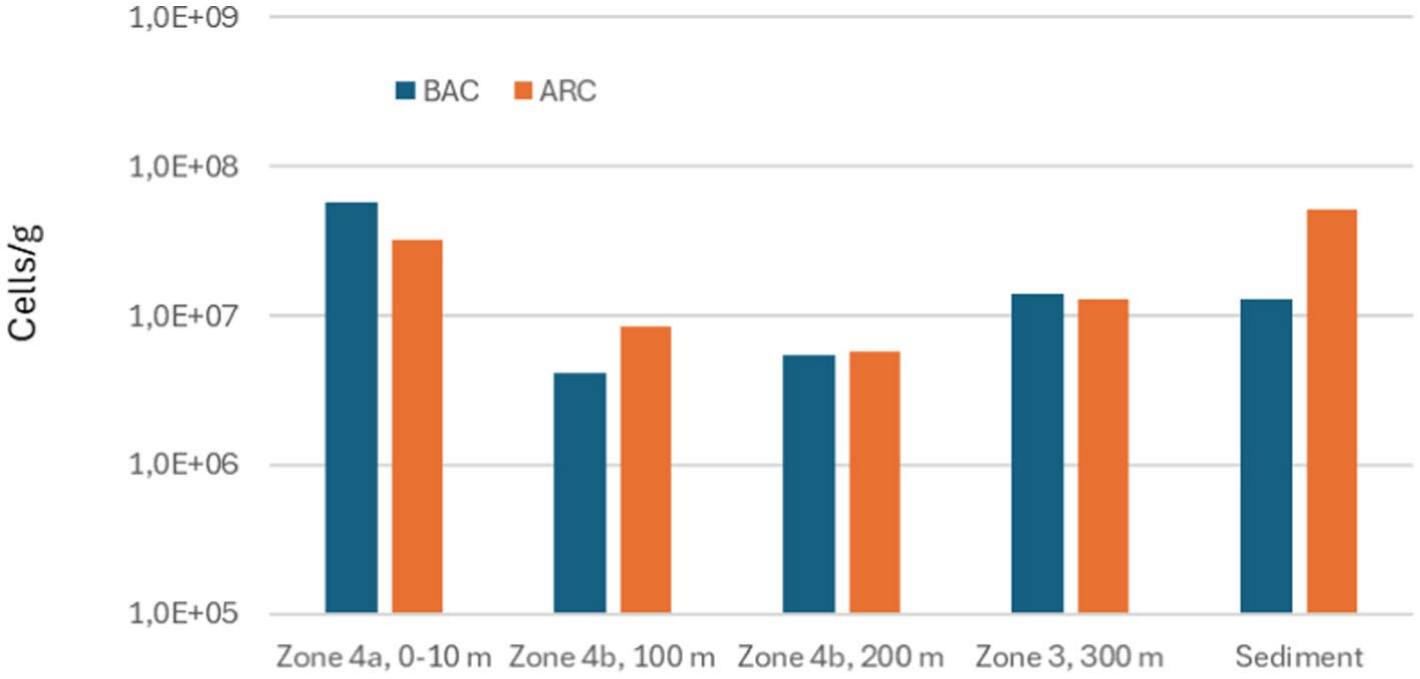
Total numbers of Bacteria (BAC) and Archaea (ARC) measured in scale from different zones on seabed chain 16, sampled in 2023.

The total numbers of *Bacteria* and *Archaea* measured in deposits from the different zones of the seabed chains and in reference sediment are illustrated in Figures 8–10.

Total numbers of Bacteria in the samples varied between 3.0×10^6^ and 1.1×10^8^ Bacteria/g and total numbers of Archaea varied between 5.1×10^6^ and 7.7×10^8^ Archaea/g. Comparing the results from seabed chains 8 and 16, both sampled in 2023, there is some variation between the two chains: For example, the sample from zone 4b (100 m) on seabed chain 8 contained much higher numbers of microorganisms (4.0×10^7^ Bacteria/g and 7.7×10^8^ Archaea/g) than the similar sample from seabed chain 16 (4.1×10^6^ Bacteria/g and 8.5×10^6^ Archaea/g). This relatively large difference between samples retrieved from similar zones on chains with identical operational history may be linked to analytical bias caused by small sample sizes (about 1 g of deposit was used for DNA extraction, see also Materials and Methods), and spatial heterogeneity of deposits on the individual chain links (see Figures 5, 6). It is clear, however, that both *Bacteria* and *Archaea* were abundant in all the samples from both 2019 and 2023.

**Figure 11:**
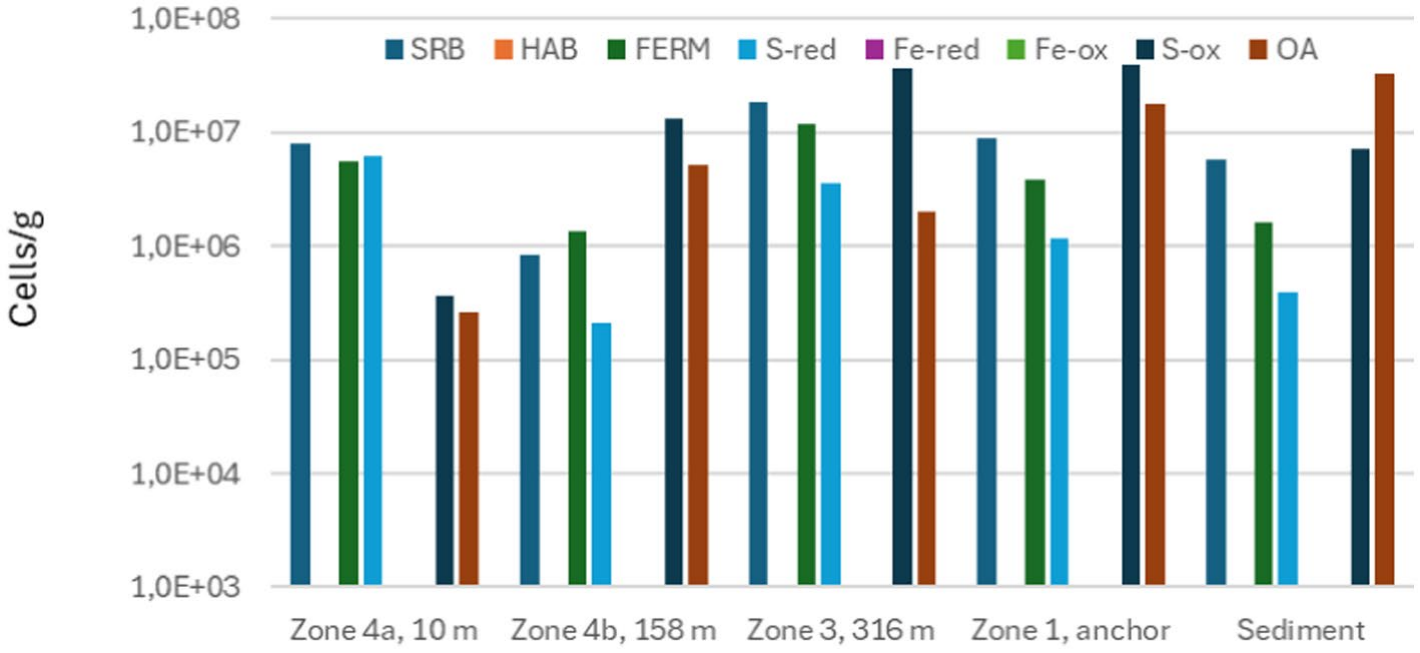
Total numbers of sulfate-reducing bacteria (SRB), homoacetogenic bacteria (HAB), fermenting bacteria (FERM), sulfur-reducing bacteria (S-red), iron-reducing bacteria (Fe-red), iron-oxidizing bacteria (Fe-ox), sulfur-oxidizing bacteria (S-ox), and aerobic bacteria other than FE-ox and S-ox (OA) measured in scale from different zones on seabed chain 1, sampled in 2019.

**Figure 12:**
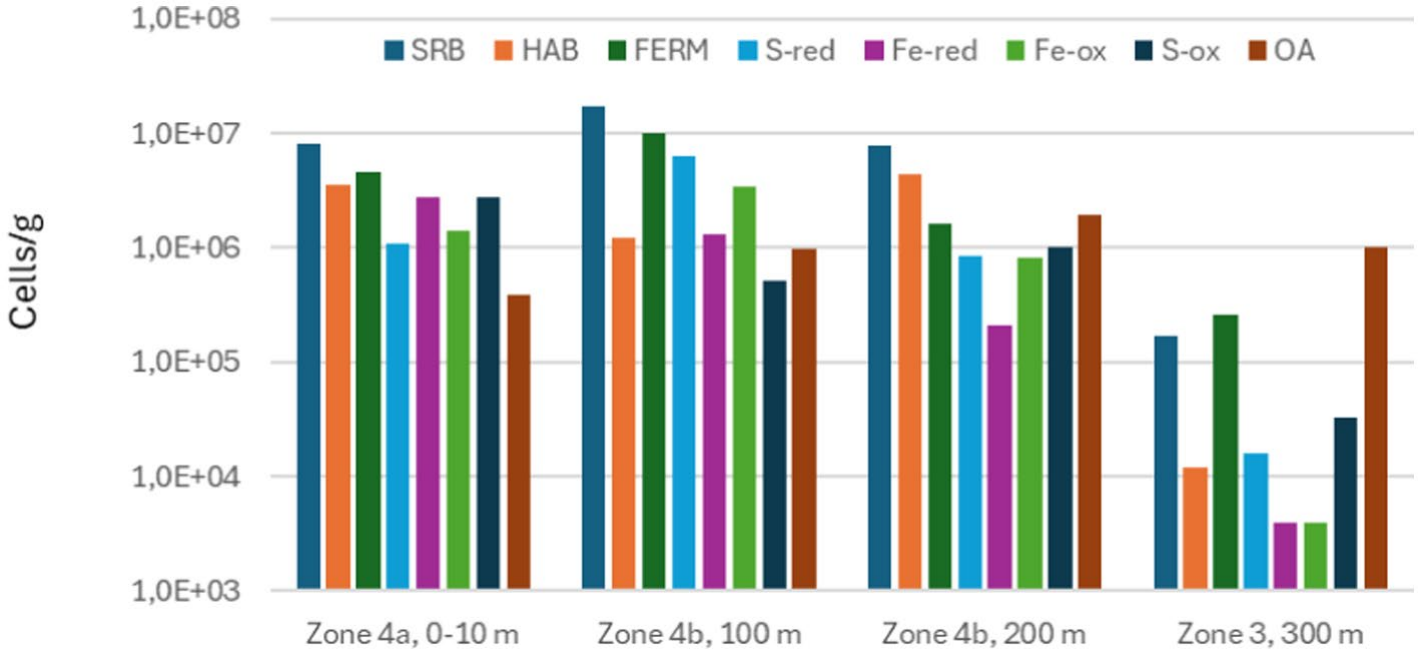
Total numbers of sulfate-reducing bacteria (SRB), homoacetogenic bacteria (HAB), fermenting bacteria (FERM), sulfur-reducing bacteria (S-red), iron-reducing bacteria (Fe-red), iron-oxidizing bacteria (Fe-ox), sulfur-oxidizing bacteria (S-ox), and aerobic bacteria other than Fe-ox and S-ox (OA) measured in scale from different zones on seabed chain 8, sampled in 2023.

**Figure 13:**
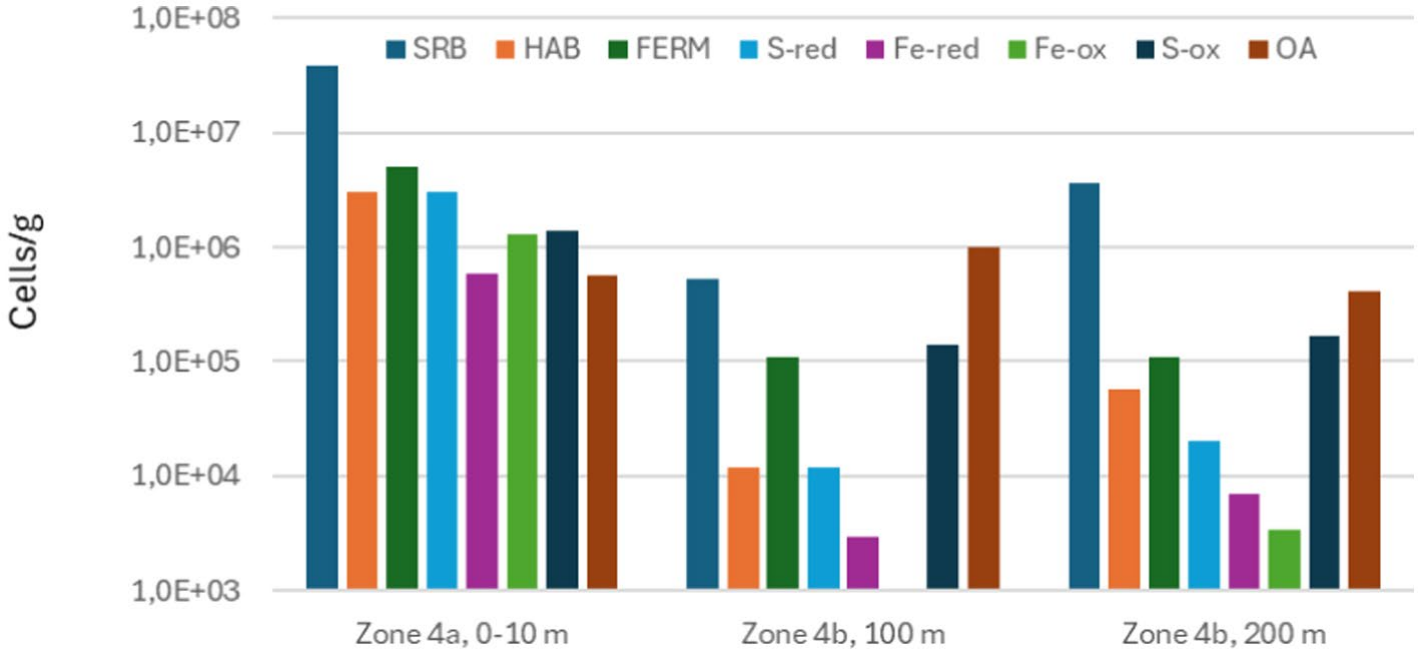
Total numbers of sulfate-reducing bacteria (SRB), homoacetogenic bacteria (HAB), fermenting bacteria (FERM), sulfur-reducing bacteria (S-red), iron-reducing bacteria (Fe-red), iron-oxidizing bacteria (Fe-ox), sulfur-oxidizing bacteria (S-ox), and aerobic bacteria other than FE-ox and S-ox (OA) measured in scale from different zones on seabed chain 16, sampled in 2023.

The phylogenetic information obtained by sequencing 16S rRNA was converted into functional information by considering the most likely metabolism of the identified genera or species. The result of this is shown in Figures 11–13.

Figure 11 shows that in 2019, seabed chain samples from Zone 4 included SRB, MEA, S-red, SOB and OA, indicating that the major microbiological metabolisms at this time were S-cycling (driven by SRB, S-red and SOB), anaerobic degradation of organic material, methanogenesis, and aerobic respiration. The localized corrosion observed during this time may have been linked to one or more of several microbial processes: In anaerobic zones, SRB and S-red will generate hydrogen sulfide (H_2_S) as major products of their metabolism, which may cause corrosion (Hesketh et al., 2021). Acidification and increased CO_2_ concentrations caused by fermentation and general degradation of organic material may also contribute (Javidi et al., 2020), as well as cathodic depolarization caused by hydrogen consumption by, e.g., SRB (Permeh et al., 2020). Certain methanogens and SRB have even been shown to live primarily by harvesting electrons from the steel surface, directly causing localized corrosion (Enning et al., 2012; Lahme et al., 2021). At the water/sediment or water/deposit interface where oxygen is present, H_2_S and other reduced sulfur compounds can be oxidized by SOB, which will decrease local pH, and thus may contribute to local corrosion rates (Rao and Mulky, 2023). H_2_S oxidation may also result in formation of corrosive products such as elemental sulfur or polysulfides on the steel surface (Erfanian et al., 2022).

By 2023, with 3 more years on the seabed and 1 year after the chains were lifted into the water column again, microbial functional diversity in the deposits had increased, as IRB, IOB, and HAB were now present in addition to the previously detected groups (Figures 12, 13). All these three groups may directly or indirectly contribute to corrosion processes at the metal surface: IOB and IRB are directly involved in biogeochemical cycling of iron and may contribute to both formation and destabilization of iron-containing minerals, including green rust (Philips et al., 2018; Duboscq et al., 2022; Ona-Nguema et al., 2004), and some HAB have been shown to be able to harvest energy in the form of electrons from the metal surface (Mand et al., 2014; Kato et al., 2014). As discussed above, data shown in Table 4 indicates the chemical composition of the deposits had become more complex in 2023 compared to 2019, and it is likely that this is linked to the increased functional diversity of the microbial community.

## 4 Discussion

During the initial 19 years in operation (1997–2016), the seabed chains appear to have been relatively unaffected by corrosion in all zones (data presented in Gabrielsen et al., 2018), but the combined corrosion, chemical, and microbiological data described above, suggests that several corrosion phenomena have been at play since then:

Resting on the sediment from 2016 to 2019 appear to have induced significant local corrosion on parts of the seabed chain with corrosion rates as high as 1.3 mm/year. During this time period, it is likely that exposing the seabed chain to anaerobic sediment which is relatively rich in nutrients compared to the overlying water has induced rapid growth of the types of microorganisms represented in Figure 11 on the steel surface, causing accelerated local corrosion due to one or more of the mechanisms described above (acidification, cathodic depolarization, electron harvesting from the metal, or formation of metabolic products such as H_2_S, CO_2_, or sulfur compounds that may induce corrosion).

Subsequently, corrosion data from the chains that were retrieved in 2023, where chains 8 and 16 had spent another 3 years on the seabed and 1 year lifted into the water column, did not indicate significant growth of pits since 2019. This indicates that local corrosion in zone 4 had slowed down already while the seabed chains were still partly buried in the sediment between 2019 and 2022. Further, no evolution in local corrosion had taken place in zone 4 between 2022 and 2023 when the chain was re-lifted into the water column.

This corrosion development is interesting in the light of the chemical and microbiological data presented above. Thus, while the observed local corrosion appeared to slow down between 2019 and 2023, the microbial community in the deposits on the seabed chains clearly became more diverse, including the emergence of new metabolic groups that are thought to be potentially corrosive (IRB, IOB and HAB). At the same time, the chemical data suggested a more dynamic cycle of corrosion products in 2023 than in 2019.

A likely explanation for this decoupling of corrosion, chemical and microbiological data, may be formation of protective layers in the deposits. Thus, the SEM/EDX analysis clearly showed that distinct layers of calcium-minerals (most likely calcium carbonate) had formed at the deposit/water interface by 2023. Such layers may efficiently inhibit the diffusional supply of dissolved nutrients to the microorganisms at the steel surface, slowing down metabolic rates and thus decreasing the rates of MIC. The much lower nutrient content in the seawater compared to the sediment environment may further have restricted microbial activity (and thus MIC) once the seabed chains were lifted back into the water phase again in 2022. In addition, both the surface layers of calcium carbonate and deeper layers of Fe-minerals (FeS, magnetite, etc.) formed at low redox zones closer to the steel/scale interface may have formed actual protective layers against MIC and chemical corrosion. In this regard, it is interesting to note that all the ingredients needed to create the self-sustaining cathodic/anodic corrosion described by Refait et al. (2016), i.e., SRB, green rust, magnetite, iron sulfide, and calcium carbonate, were observed in the deposits from 2023.

## 5 Conclusion

This study provides insights into the corrosion mechanisms on offshore seabed chains and potential effects of operational actions. It shows how localized corrosion caused by MIC includes a complex microbial ecosystem that vary spatially and temporally, and is directly affected both by the environmental conditions, in this case resting on the seabed versus placed in the water column, and by local conditions such as scale deposition and insulating/diffusional barriers inside the deposits. Specifically, the study demonstrates how localized corrosion caused by MIC may not be predicted solely based on microbiological measurements but should also include a detailed knowledge of the mineral structure of the deposits.

The study demonstrates that effective integrity management strategies should consider the effects of operational disruptions and environmental transitions on corrosion phenomena such as MIC and adds new aspects to the complexity of service life estimations of offshore seabed chains.

## Data Availability

The original contributions presented in the study are included in the article/supplementary material, further inquiries can be directed to the corresponding author.

## Footnotes

1 Data from Kystinfo, https://kart.kystverket.no/.
